# Mental Fatigue and Resistance Exercise: A Systematic Review and Meta‐Analysis Including GRADE Qualification

**DOI:** 10.1002/ejsc.70194

**Published:** 2026-05-21

**Authors:** Luiz José Frota Solon‐Júnior, Leonardo de Sousa Fortes, Gustavo Vasconcelos, Nadia Abasrashid, Sandro Bartolomei, Samuele Maria Marcora, Dalton de Lima‐Junior

**Affiliations:** ^1^ Federal University of Paraíba Joao Pessoa Brazil; ^2^ Regional University of Cariri Iguatu Brazil; ^3^ Leão Sampaio University Center Juazeiro do Norte Brazil; ^4^ Federal University of Minas Gerais Belo Horizonte Brazil; ^5^ Federal University of São Paulo São Paulo Brazil; ^6^ University of Bologna Bologna Italy; ^7^ Faculty of Health, School of Exercise and Nutrition Sciences Queensland University of Technology Brisbane Australia; ^8^ Department of Sport and Exercise Psychology University of Potsdam Potsdam Germany; ^9^ Department of Theoretical and Applied Sciences eCampus University Novedrate Italy; ^10^ LUDES University of Applied Sciences Institute Lugano Switzerland

**Keywords:** brain, cognitive effort, mental demand, physical exercise, resistance training

## Abstract

The recent increase in randomized controlled trials evaluating the effects of mental fatigue (MF) on RE warrants an update of the available meta‐analytical evidence. This study presents the results of a comprehensive systematic review and meta‐analysis, including GRADE qualification, examining the effects of MF on RE across different subgroups. We included only randomized controlled trials involving healthy human participants, a high‐demand cognitive task requiring cognitive effort (e.g., Stroop test), a low‐demand or passive control condition (e.g., watching documentaries), and a resistance exercise performance task assessing volume. A total of 11 studies reported 14 comparisons involving more than 205 participants, providing moderate‐level evidence. The random‐effects meta‐analysis revealed a significant mean negative effect of cognitive effort on RE volume (*g* = −0.39 and *p* < 0.01). Subgroup analyses suggested that multijoint exercises may be more susceptible to impairment (*g* = −0.45 and *p < 0.01*) than single‐joint exercises (*g* = −0.20 and *p = 0.09*). The magnitude of impairment appeared larger at moderate‐intensity loads (60%–79% 1RM and *g* = −0.56) relative to low‐intensity (*g* = −0.40) and bodyweight conditions (*g* = −0.25). Similarly, the effect appeared greater in high training volume conditions (*g* = −0.54) compared to moderate volume (*g* = −0.37). Overall, although this review confirms the negative impact of MF on RE volume, the findings should be interpreted with caution due to low‐quality evidence.

## Highlights

- Mental fatigue has a small‐to‐moderate negative effect on resistance exercise volume (Hedges' *g* = −0.39 and 95% CI: −0.55, −0.23), but this is based on low‐quality evidence and should be interpreted cautiously.
- Mental fatigue significantly impairs multijoint exercises (*g* = 0.45 and *p* < 0.01), but not single‐joint exercises (*g* = 0.20 and *p* = 0.09), and its detrimental effect appeared larger at moderate intensities (60%–79% 1RM and *g* = −0.56) relative to low‐intensity (*g* = −0.40) and bodyweight conditions (*g* = −0.25), suggesting that complexity and load intensity may modulate susceptibility to this phenomenon.
- Mental fatigue has a greater detrimental effect on performance in exercise sessions with higher relative volume [repetitions × sets × load intensity (%1RM)] (*g* = −0.54) than in sessions with lower relative volume (*g* = −0.37).

## 1 Introduction

Engaging in cognitive activity may require low or high mental demand and consequently cause a psychobiological state called mental fatigue (MF) that presents symptoms, such as tiredness, lack of energy, increased perception of effort, and reduced cognitive ability, resulting in a performance reduction in a subsequent task (Brown et al. 2020; Pageaux et al. 2015; Smith et al. 2018). Although research in this field has shown contradictory results (Holgado et al. 2023, 2025), these symptoms appear to impair physical performance by reducing cognitive and physical abilities (Fortes et al. 2020; Marcora et al. 2009; Penna et al. 2021). In daily life, MF can arise from various activities, such as studying, working, driving, cycling, watching movies, or using smartphones for social media (Gantois et al. 2021; Hockey and Earle 2006; Simon et al. 2011; Zeuwts et al. 2021).

MF may impair higher‐order cognitive control (Lorist et al. 2005) and reduce the ability to manage attentional control and the encoding and storage of relevant information, leading to less efficient behavior and an increased perceived cost‐future reward relationship in a given task (Kok 2022). Given these cognitive impairments, MF is hypothesized to negatively impact resistance exercise performance, particularly by reducing exercise volume (Alix‐Fages, González‐Cano, et al. 2023a; De Lima‐Junior et al. 2024; Gantois et al. 2021), a key determinant of long‐term training adaptations such as muscular hypertrophy and strength (Figueiredo et al. 2018). Because long‐term training outcomes rely on the quality and consistency of individual sessions, any factor that acutely reduces exercise volume may diminish training‐induced adaptations over time (Figueiredo et al. 2018; Li et al. 2018). Consequently, any factor that systematically reduces the volume achieved during resistance exercise sessions may compromise long‐term training outcomes.

Although MF has been extensively investigated in endurance exercise (Habay et al. 2023), its effects on resistance exercise have only recently received attention. A prior meta‐analysis by Alix‐Fages, Grgic, et al. (2023b) reported significant negative effects of MF on resistance exercise volume, particularly in the number of repetitions for upper (effect size [ES]: −0.41 and 95% confidence interval [CI] [−0.70, −0.12]) and lower limbs (effect size [ES]: −0.39 and 95% CI [−0.75, −0.04]); however, methodological limitations might constrain the reliability of these conclusions and warrant an updated synthesis (Alix‐Fages, Grgic, et al. 2023b). Although the review highlights important aspects of MF and RE, it lacks prospective registration, assessment of publication bias, and a GRADE evaluation of the evidence—components widely regarded as essential in contemporary systematic review methodology (Higgins et al. 2019; Moher et al. 2009; J. A. C. Sterne et al. 2011). Furthermore, five recent studies were not included in the analysis (Alix‐Fages, González‐Cano, et al. 2023a; Cuchna et al. 2024; De Lima‐Junior et al. 2024; De Salles Painelli et al. 2024; Solon‐Júnior et al. 2026) that might substantively alter the pooled effect estimate.

Additionally, two key questions were addressed and are directly examined in the present meta‐analysis through planned subgroup analyses. First, it is unclear whether the effect magnitude of MF varies with the intensity‐load zone (% of 1‐repetition maximum—RM) of RE (i.e., low‐, moderate‐, and high‐intensity load and bodyweight exercises). Although one study (De Lima‐Junior et al. 2024) has examined MF effects at different intensities (i.e., 50%, 70%, and 90% of 1RM), no quantitative synthesis across studies has been performed. Thus, given that RT adaptations may vary with different volume‐matched loads (Carvalho et al. 2022; Schoenfeld et al. 2017), a meta‐analysis with subgroup analysis is needed to examine the effects of MF across RE protocols utilizing very low and low (< 30%–59% of 1RM), moderate (60%–79% of 1RM), and high (≥ 80% of 1RM) intensity‐loads as well as bodyweight exercises.

Second, it remains uncertain whether exercise complexity modulates susceptibility to MF. Tasks inducing MF impair performance mainly through cortical alterations that increase the perception of effort rather than through peripheral muscular mechanisms (Marcora et al. 2009). Multijoint movements (e.g., squat and bench press) impose substantially greater demands on central executive functions, including motor planning, intersegmental coordination, attentional allocation, and sensorimotor integration, compared to single‐joint movements (e.g., biceps curl and leg extension) (Seidler et al. 2004). Therefore, we hypothesized that multijoint exercises would be more vulnerable to mental fatigue‐induced performance decrements and examined this hypothesis through a planned subgroup analysis.

Third, and perhaps most importantly, the current systematic review on this topic lacks a strength‐of‐evidence assessment such as the GRADE approach (Alix‐Fages, Grgic, et al. 2023b). For these reasons, although the authors from the previous systematic review made a great contribution to the literature, a comprehensive and up‐to‐date systematic review with meta‐analysis using GRADE is essential to enhance the clarity of findings (Garner et al. 2016) and inform trainers, practitioners, and researchers about the effects of MF on RE. Also, a more thorough documentation of these important features sets the stage for continued updates from this field, using best‐practice procedures for analysis and reporting.

Then, the primary aim of this systematic review and meta‐analysis was conducted to provide an updated, comprehensive synthesis of MF effects on RE volume [that is, number of repetitions and total training volume load (TTV; number of repetitions x number of sets x load)] in healthy individuals, incorporating recent evidence, rigorous publication bias assessment, and GRADE evidence evaluation. Secondary aims were to examine potential moderators through subgroup analyses of exercise complexity (single‐joint vs. multijoint) and intensity‐load zones. These findings will clarify whether MF should be avoided before RE sessions and inform evidence‐based recommendations for practitioners. Thus, the research question for this systematic review was as follows: Does MF induced by previous cognitive effort reduce subsequent RE volume in apparently healthy adults?

## 2 Methods

### 2.1 Experimental Approach to the Problem

For this systematic review and metanalysis, including GRADE qualification, we investigate the effects of MF on dynamic RE volume. We followed the methods established prior to the review and adhered to the guidelines of the Preferred Reporting Items for Systematic Reviews and Meta‐Analyses (PRISMA) for systematic reviews (Moher et al. 2009). Additionally, we prospectively registered the review in PROSPERO (ID: CRD420251003971).

### 2.2 Criteria for Eligibility

The inclusion criteria for this systematic review are based on PICOS (Population, Intervention, Comparison, Outcome, and Study design) standards: (a) to be performed in healthy humans; (b) a high cognitive demands task intervention that requires sustained cognitive engagement, such as response inhibition, selective attention, or continuous performance monitoring; (c) a comparison group or experimental condition with low cognitive demands or passive control condition (e.g., watching documentaries and seated rest); (d) the outcome variable must be related to dynamic RE volume; and (e) a randomized controlled trial.

### 2.3 Search Strategy and Study Selection

**FIGURE 1:**
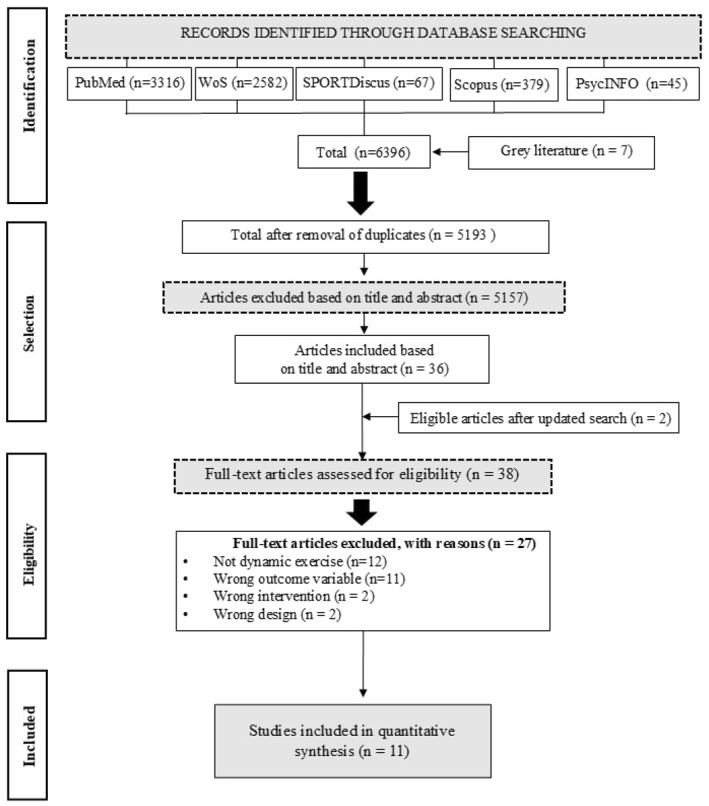
PRISMA flowchart. The selection process of 11 studies included in the systematic review based on PRISMA (Preferred Reporting Items for Systematic Reviews and Meta‐Analyses) recommendations.

The search strategy is described in Figure 1. We searched the PubMed, Web of Science (WoS), SPORTDiscus, Scopus, and PsycINFO databases. The earliest available date up to November 2024 was considered in the process, with no language limitation. An updated search was conducted in February 2026 to ensure the inclusion of the most recent evidence. Search terms included combinations of MF and RE description [that is, (mental* fatigue* OR mental* effort* OR mental* exert* OR mental* exhaust* OR mental* strain* OR cognitive* fatigue* OR cognitive* effort* OR cognitive* exert* OR cognitive* exhaust* OR cognitive* strain* OR self‐control* depletion OR ego depletion) AND (resist* training OR resist* exercise OR strength* training OR strength* exercise OR muscular endurance* OR weight training OR weight exercise OR weightlifting OR power training OR power exercise OR circuit training OR circuit exercise OR plyometric training OR plyometric exercise)]. Search strategies were modified to meet the specific requirements of each database. The search was not limited to specific years. To minimize publication bias, we employed a gray literature search strategy encompassing: (a) backward citation searching of reference lists from all included studies and (b) systematic searching of the Open Science Framework (OSF), a free open‐source online platform hosting preprints and unpublished research. After identification, we first screened articles based on their titles and abstracts. Then, a full‐text evaluation was performed according to the PICOS criteria. Two researchers (LJFSJ and NA) performed the previous steps, and conflicts were solved by a third party (DLJ). For this review, RE was operationalized as isotonic (concentric‐eccentric) muscle contractions performed against an external resistance, including free weights, weight machines, resistance bands, and body weight. Isometric contractions and isokinetic testing protocols were excluded.

### 2.4 Risk of Bias Assessment

Each study's methods were evaluated using the TESTEX (Smart et al. 2015). Several tools are available to measure and report the quality of randomized controlled trials. However, in practice, some criteria, such as participant or researcher blinding, are sometimes unfeasible. In this case, TESTEX is the most appropriate tool to assess randomized controlled trials in physical exercise. The tool comprises 12 items, with scores ranging from 0 to 15: five points for quality and 10 for reporting. Each article was evaluated by two researchers (LJFSJ and GV), and a third researcher resolved disagreements.

### 2.5 Quality of Evidence

The overall quality of the evidence for the outcome was evaluated using the Grading of Recommendations, Development and Evaluation (GRADE) system (Higgins et al. 2019). The following five factors were considered for classifying the quality of the evidence, where for each factor not met, the quality of evidence was reduced by one level (from high to moderate and low or very low) (Higgins et al. 2019): risk of bias (> 25% of the trials included in the comparison were classified as high risk of bias), inconsistency (I^2^ > 50%), indirectness (> 50% of the participants were not related to the trial's target audience and differences in interventions), imprecision (< 400 participants in the comparison for continuous outcomes and > 300 participants for categorical outcomes), and publication bias (evaluated using a funnel plot when > 10 trials in the same comparison or an extensive search on databases and gray literature). Single‐trial comparisons (< 400 participants for continuous outcomes and < 300 participants for dichotomous outcomes) were considered to be inconsistent and imprecise, providing “low‐quality evidence”, which could be downgraded to “very low‐quality evidence” if limitations were identified regarding risk of bias (Higgins et al. 2019). The quality of the evidence was categorized as follows. Evidence was high quality if the results were consistent across ≥ 75% of participants, with low risk of bias, without publication bias, and with consistent, direct, and precise data; further research is unlikely to change the estimate or confidence in these results. Evidence was of moderate quality when one of the five classification factors above was not met; further research is likely to change the estimated effect and impact confidence in the effect. The evidence was of low quality because two of the five classification factors were not met; future research is likely to change the estimated effect and will have a significant impact on confidence in the effect. The evidence was very low quality when three of the five classification factors were not met; any estimate of effect is very uncertain.

### 2.6 Data Analysis and Bias of Publication

The effect size for each study was calculated as the difference in performance (i.e., dynamic RE volume) between the experimental (i.e., MF) and control conditions. The mean difference and 95% confidence interval (CI) were calculated to measure the intervention effect on continuous outcomes. Standard deviations for the analysis were extracted using the methods described in the Cochrane Handbook (Higgins et al. 2019). The meta‐analysis was conducted using RevMan software (version 5.4) with a random‐effects model (standardized mean difference—SMD and Hedge's g adjusted for small sample bias) to calculate the pooled treatment effect with a 95% confidence interval as we anticipated that the selected studies varied in design, participants, and interventions, suggesting that these studies may not share the same true effect size. Thus, for between‐participants trials, we calculated Hedges' g (bias‐corrected SMD) using postintervention means and pooled SD. For within‐participants (crossover) trials, we computed standardized mean change with raw‐score standardization (SMCR) for the MF versus control difference. We converted it to Hedges' g, incorporating the within‐person correlation between conditions in the sampling variance (Morris and DeShon 2002). Missing means and standard deviations were calculated based on t‐values and *p*‐values for mean differences (Higgins et al. 2019) or obtained from figures using the WebPlotDigitizer (https://apps.automeris.io/wpd/). According to Hedge's g guidelines, SMD values of 0.2, 0.5, and 0.8 indicate small, moderate, and large effect sizes, respectively. Statistical significance was set at *p* ≤ 0.05. Statistical heterogeneity was assessed using the I^2^ statistic and visual inspection of the forest plot.

To further characterize uncertainty in the context of between‐study heterogeneity, a 95% prediction interval (PI) was calculated for the primary meta‐analysis following the formula (Borg et al. 2024; Riley et al. 2011):

$$\text{SDPI}=\sqrt{(\text{SE}\_{\hat{\mu }}^{2}+{\hat{\tau }}^{2})}\;\hat{\mu }\pm \;t\_\left(K-2\right)\left(1\;-\;\alpha /2\right)\times \text{SD}\_\text{PI}$$

where:

- $\hat{\mu }$ = the pooled effect
- t_(K−2) (1 − α/2) = right tail α/2 quantile of a t‐distribution with K−2 degrees of freedom, where K is the number of estimates in the analysis
- SE_$\hat{\mu }$ = the standard error of the pooled effect
- ${\hat{\tau }}^{2}$ = estimated between‐study heterogeneity variance
- SD_PI = standard deviation of the prediction interval

Publication bias was systematically evaluated using multiple complementary methods, including visual inspection of funnel plot asymmetry, trim‐and‐fill adjustment procedure (Duval and Tweedie 2000a, 2000b), Egger's regression test (Egger et al. 2002), and Begg and Mazumdar's rank correlation test (Begg and Mazumdar 1994). The funnel plot serves as the primary graphical tool for detecting publication bias by examining the relationship between study precision (standard error) and effect size magnitude (J. A. Sterne and Egger 2001). To complement the visual assessment of funnel plot asymmetry, we conducted Egger's regression test, a formal statistical procedure that quantifies asymmetry through weighted linear regression. As a complementary nonparametric approach to assessing publication bias, we employed Begg and Mazumdar's rank correlation test (Begg and Mazumdar 1994), which evaluates the correlation between standardized effect sizes and their sampling variances using Kendall's tau rank correlation coefficient. Finally, to quantitatively estimate the potential impact of publication bias on the combined effect size, we employed the trim‐and‐fill method (Duval and Tweedie 2000a, 2000b), an iterative nonparametric procedure designed to: (1) identify and “trim” asymmetrically distributed studies, (2) re‐estimate the combined effect size based on the trimmed dataset, and (3) “fill” the funnel plot with imputed studies to achieve symmetry. Additionally, a test of excess significance (Ioannidis and Trikalinos 2007) was conducted to assess whether the number of statistically significant results among the included studies exceeded what would be expected given the estimated statistical power of the individual studies.

## 3 Results

### 3.1 Included Studies

In the analysis, we identified 6396 studies, of which 5193 remained after duplicate removal. Following the screening of 5193 abstracts, 36 studies were selected for full‐text review and only 10 met the required standards. An updated search conducted in February 2026 identified additional records, from which two studies were selected for full‐text assessment. After careful evaluation against our eligibility criteria, one of these newly identified studies met all inclusion requirements. In total, 11 studies were included in the final analysis and 27 studies were excluded during the full‐text review stage (see Figure 1). The rationale for excluding each study is detailed in Supporting Information S1.

### 3.2 Characteristics of the Studies Included

A detailed description of the studies is presented in Table 1. Study details and outcomes were divided into (a) study; (b) number of participants (N); (c) participants; (d) design; (e) cognitive task; (f) control task; (g) task duration; (h) manipulation check; and (i) warm‐up and physical task. All the data presented in Table 2 were independently extracted from studies by two review authors (LJFSJ and DLJ). Examination of funding sources, author affiliations, and conflict of interest statements revealed that all included studies reported no conflicts of interest.

**TABLE 1:** TESTEX scale's score.

Study	Methodological quality of studies according to TESTEX scale.^a^												Total
	#1	#2	#3	#4	#5	#6	#7	#8	#9	#10	#11	#12	
Brown et al. (2021)	0	0	1	1	1	2	1	2	1	1	1	1	12
De Lima‐Junior et al. (2024)	1	1	1	1	0	3	1	2	1	1	1	1	14
Dorris et al. (2012)^b^	0	0	0	1	0	2	1	2	1	1	1	1	10
Dorris et al. (2012)^c^	0	0	0	1	0	2	1	2	1	1	1	1	10
Gantois et al. (2021)	0	0	1	1	1	2	1	2	1	1	1	1	12
Head et al. (2016)	0	0	0	1	1	2	1	2	1	1	1	1	11
Queiros et al. (2021)	1	1	0	1	0	2	1	2	1	1	1	1	12
Graham et al. (2017)	1	0	0	1	0	2	1	2	1	1	1	1	11
De Salles Painelli et al. (2024)	1	1	0	1	0	2	1	2	1	1	1	1	12
Alix‐Fages, González‐Cano, et al. (2023a); Alix‐Fages, Grgic, et al. (2023b)	0	1	1	1	1	2	1	2	1	1	1	1	13
Cuchna et al. (2024)	0	0	0	1	0	1	1	2	1	1	1	1	9
Solon‐Júnior et al. (2026)	1	1	0	1	0	3	1	2	1	1	1	1	13

Abbreviations: #1 = Eligibility criteria specified, #2 = randomization specified, #3 = allocation concealment, #4 = groups similar at baseline, #5 = blinding of assessor (for at least one key outcome), #6 = outcome measures assessed in 85% of patients, #7 = intention‐to‐treat analysis, #8 = between‐group statistical comparisons reported, #9 = point measures and measures of variability for all reported outcome measures, #10 = activity monitoring in control group, #11 = relative exercise intensity remained constant, and #12 = exercise volume and energy expenditure.

^a^ The points were awarded ONLY if the criteria was clearly satisfied.

^b^ Experiment 1.

^c^ Experiment 2.

**TABLE 2:** Overview of studies' characteristics.

Study	Participants	Design	Cognitive task	Control task	Task duration	MC	Warm up + physical task
Brown et al. (2021)	Physically active young adults (*N* = 10)	Within	Incongruent Stroop task	Documentary	10‐min	↑ RPME	Warm up (1 set of 10 rep; 7.73 kg) + biceps curl (50% 1RM) until disengagement.
De Lima‐Junior et al. (2024)	Resistance‐trained young men (*N* = 18)	Within	Incongruent Stroop task	Documentary	30‐min	↑ RPME	Warm up (5‐min on cycle ergometer + 2 sets of 6 rep at 50% and 70% of 1 RM) + half back‐squat (50%, 70%, and 90% 1RM) until disengagement
Dorris et al. (2012)—Experiment 1	Competitive rowers (*N* = 24)	Within	Difficult arithmetic/balancing task.	Easy arithmetic/balancing task	Irregular time	= RPME	Warm up not mentioned + perform push‐ups until disengagement
Dorris et al. (2012)—Experiment 2	Hockey and rugby players (*N* = 24)	Within	Difficult arithmetic/balancing task.	Easy arithmetic/balancing task	Irregular time	↑ RPME	Warm up not mentioned + perform sit‐ups until disengagement
Gantois et al. (2021)	Recreationally strength‐trained young adults (*N* = 16)	Within	Social media on smartphones	Documentary	30‐min	↑ RPME	Warm up (2 sets of 15 rep at 50% and 80% of 1 RM) + half back‐squat [80%15RM (∼50% 1RM)] until disengagement
Head et al. (2016)	Physically active young adults (*N* = 18)	Within	Vigilance test	Documentary	52‐min	↑ RPME	Warm up (5‐min; standardized dynamic) + blocks of 5 pull‐ups, 10 push‐ups, and 15 unweighted squats in 20‐min
Queiros et al. (2021)	Resistance‐trained young adults (*N* = 9)	Within	Stroop task	Documentary	30‐min	↑ RPME	Warm up (5‐min on cycle ergometer + 1 set of 10 rep at 60% of 1RM) + squat (70%1RM) until disengagement
Graham et al. (2017)	Physically active young adults (*N* = 50)	Between	Incongruent Stroop task	Congruent Stroop task	5‐min	↑ RPME	Warm up (5‐min on cycle ergometer + 1 set of 10 rep at 25% of 1RM) + bench press (60% of 1RM), and leg extension (40% 1RM) until disengagement
De Salles Painelli et al. (2024)	Resistance‐trained young adults (*N* = 12)	Within	Stroop task	Seated	30‐min	↑ RPME	Warm up (5‐min on cycle ergometer + 1 set of 8 rep at ∼50% of 1RM) + bench press (70% 1RM) until disengagement
Alix‐Fages, González‐Cano, et al. (2023a); Alix‐Fages, Grgic, et al. (2023b)	Resistance‐trained young adults (*N* = 21)	Within	Incongruent Stroop task and social media on smartphones	Documentary	30‐min	↑ RPME	Warm up (jogging, dynamic stretching for elbow and shoulder mobility, and 3 sets of 10, 5, and 3 repetitions with 30%, 45%, and 60% of 1RM, respectively) + bench press (65% 1RM) until disengagement
Cuchna et al. (2024)	Resistance training experience (*N* = 7)	Within	Stroop task	Watching a segment of Seinfeld	30‐min	↑ RPME	Warm up not mentioned + leg press (50% 1RM) until disengagement
Solon‐Júnior et al. (2026)	Resistance‐trained young men (*N* = 20)	Within	Stroop task	Seated	Irregular time [moderate MF (40 mm) and high MF (80 mm)]	↑ RPME	Warm up (2 sets of 3 rep; ∼45% 1‐RM) + half‐back squat (∼45% 1‐RM) until disengagement

Abbreviations: BET, brain endurance training; MC, manipulation check; RM, repetition maximum; RPME, rating of perceived mental exertion.

### 3.3 Characteristics of Participants

More than 205 participants were included across the evaluated studies. The number of participants per study ranged from seven (Cuchna et al. 2024) to fifty (Graham et al. 2017). The participants consisted of physically active individuals (Brown et al. 2021; Graham et al. 2017; Head et al. 2016) and recreationally trained adults (Alix‐Fages, González‐Cano, et al. 2023a; Cuchna et al. 2024; De Lima‐Junior et al. 2024; De Salles Painelli et al. 2024; Gantois et al. 2021; Solon‐Júnior et al. 2026; Queiros et al. 2021). We also included hockey and rugby players (Dorris et al. 2012) and competitive rowers (Dorris et al. 2012). Therefore, this suggests low variability in the characteristics of the included subjects.

### 3.4 Methods Used to Induce MF and Manipulation Checks

Regarding cognitive tasks, studies utilized social media on smartphone (Alix‐Fages, González‐Cano, et al. 2023a; Gantois et al. 2021), a congruent Stroop test (Cuchna et al. 2024; De Salles Painelli et al. 2024) and, primarily, an incongruent Stroop test (Alix‐Fages, González‐Cano, et al. 2023a; Brown et al. 2021; De Lima‐Junior et al. 2024; Graham et al. 2017; Solon‐Júnior et al. 2026; Queiros et al. 2021). Other studies used a challenging arithmetic/balance task (Dorris et al. 2012) or a vigilance test (Head et al. 2016). The duration of cognitive tasks varied significantly, ranging from five (Graham et al. 2017) to 52 min (Head et al. 2016). Only two studies reported an irregular time for cognitive tasks (Dorris et al. 2012; Solon‐Júnior et al. 2026).

Several manipulation checks were used to evaluate MF. Subjective measures were widely used; therefore, postcognitive effort data were collected from both groups (experimental vs. control). Subjective measures vary between studies, but the “M‐VAS” scale (i.e., MF visual analog scale) was widely used (Alix‐Fages, González‐Cano, et al. 2023a; Brown et al. 2021; De Lima‐Junior et al. 2024; De Salles Painelli et al. 2024; Gantois et al. 2021; Solon‐Júnior et al. 2026; Queiros et al. 2021). Other subjective measures, such as NASA‐TLX (i.e., Global workload and mental demand) (Cuchna et al. 2024; Head et al. 2016), ratings of perceived mental exertion (i.e., CR‐10 scale) (Graham et al. 2017), and a 7‐point scale (Head et al. 2016) were also used.

### 3.5 Methodological Quality of Articles

The selected studies were assessed using the TESTEX methodological quality scale. One study scored 9/15 (Cuchna et al. 2024), one study scored 10/15 (Dorris et al. 2012), two studies scored 11/15 (Graham et al. 2017; Head et al. 2016), four studies obtained 12/15 (Brown et al. 2021; De Salles Painelli et al. 2024; Gantois et al. 2021; Queiros et al. 2021), two studies scored 13/15 (Alix‐Fages, González‐Cano, et al. 2023a; Solon‐Júnior et al. 2026), and one scored 14/15 (De Lima‐Junior et al. 2024). Table 1 presents the detailed TESTEX scale scores for each study. The mean TESTEX score for the included studies was 11.58 ± 1.44 out of 15, indicating good methodological quality. To examine whether methodological quality influenced the pooled effect estimate, we conducted a sensitivity analysis excluding studies that scored ≤ 10/15 (≤ 67% of the maximum score), which represented the lower end of the quality distribution. This threshold‐based approach removed three comparisons from two studies (Cuchna et al. 2024; Dorris et al. 2012—both comparisons). The sensitivity analysis, including only studies with TESTEX > 10/15 (*n* = 9 studies and 11 comparisons), yielded results (*g* = −0.43; 95% CI: −0.63, −0.23; *p* < 0.01) that were similar to the primary analysis (*g* = −0.39; 95% CI: −0.56, −0.21; and *p* < 0.01), indicating that the findings are robust to variations in methodological quality.

### 3.6 Effect of Mental Fatigue on Resistance Exercise Volume (Overall Effect)

**FIGURE 2:**
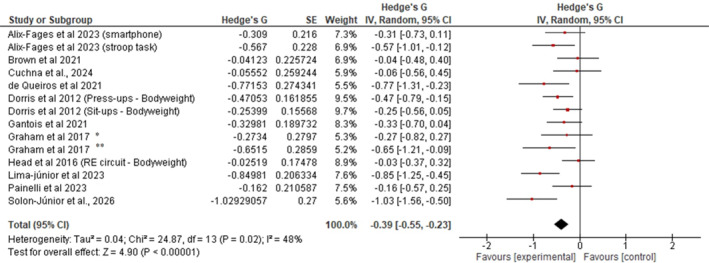
Forest plot presenting the results of the random‐effects meta‐analysis comparing the effects of MF versus control in the volume of resistance training.

The systematic search identified 14 interventions across 11 studies that examined the effects of MF on RE volume (number of repetitions or volume‐load). Although three studies (Alix‐Fages, González‐Cano, et al. 2023a; Dorris et al. 2012; Graham et al. 2017) contributed more than one comparison to the meta‐analysis, this approach was methodologically justified as each comparison involved distinct experimental conditions (e.g., different cognitive tasks or different participant populations within the same study). Therefore, this approach is consistent with Cochrane guidance for handling multiple intervention arms (Higgins et al. 2019). As shown in Figure 2, there was an overall effect of MF on resistance exercise volume [that is, number of repetitions and total training volume (TTV; number of repetitions × number of sets × load)] (standardized mean difference [SMD] = −0.39; IC 95% = −0.55 a −0.23; and *p* < 0.01), with significant heterogeneity (I^2^ = 48% and *p* < 0.05). The 95% prediction interval for the overall effect ranged from −0.861 to 0.081, indicating that while the average effect is negative and statistically significant. This interval reflects the influence of between‐study heterogeneity (*τ* ^2^ = 0.04 and I^2^ = 48%) on the expected treatment effect in future applications and should be considered alongside the confidence interval when interpreting the practical relevance of these findings (Borg et al. 2024; Riley et al. 2011).

### 3.7 Publication Bias Analysis

**FIGURE 3:**
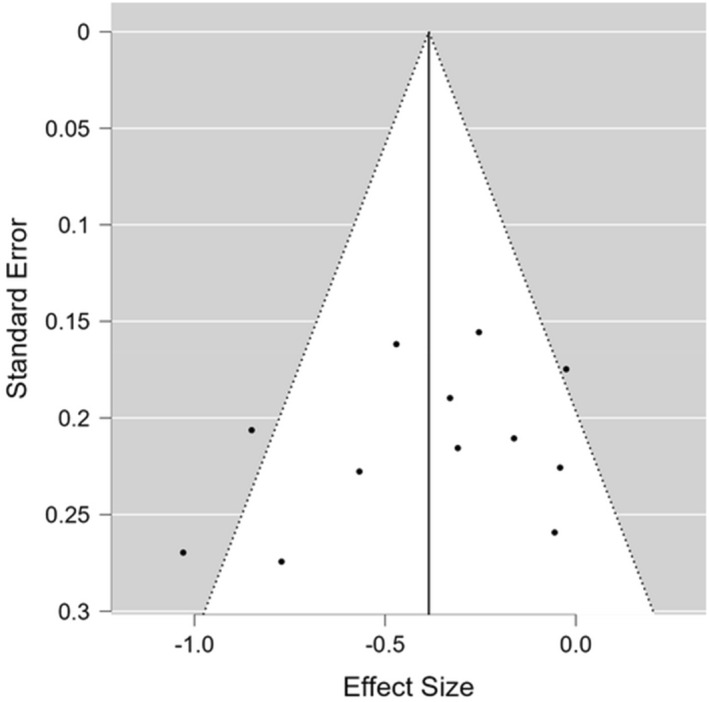
Funnel plot of included studies (*k* = 14 comparisons) examining the effects of mental fatigue on resistance exercise volume.

Visual inspection of the funnel plot revealed a relatively symmetric distribution with only a slight gap observed in the lower‐right side (Figure 3). Egger's regression analysis yielded a nonsignificant intercept (intercept = −3.88; 95% CI: −9.71, 1.94; *t* = −1.44; and *p* = 0.18), indicating that this test did not detect statistically significant asymmetry in the funnel plot. Similarly, the Begg and Mazumdar rank correlation test showed a weak inverse, nonsignificant relationship (Kendall's tau‐a = −0.23; *z* = −1.15; and *p* = 0.25) between the rankings of effect sizes and variances, providing no statistical evidence of publication bias using this nonparametric approach.

The Duval and Tweedie's trim and fill procedure was applied as a sensitivity analysis to estimate the number of potentially missing studies. The algorithm identified zero studies to be filled (*L*0 = 0). Consequently, the adjusted effect size remained identical to the observed estimate (Hedges' *g* = −0.39 and 95% CI [−0.56, −0.21]), indicating that the primary findings are robust against the “file drawer” effect. Finally, a test of excess significance (Ioannidis and Trikalinos 2007) was conducted to assess whether the number of significant results in the sample exceeded what would be expected based on the statistical power of the included studies. The observed number of significant results (*O* = 5) was highly consistent with the expected value (*E* = 5.52) (*O/E* ratio = 0.91), with a nonsignificant *p*‐value (*p* = 0.73). This lack of excess significance provides further evidence that the results have not been distorted by selective reporting of significant findings. However, given the small number of studies included in this meta‐analysis, caution is advised when interpreting these tests of small study effects and publication bias.

### 3.8 Effects of Mental Fatigue in Single and Multi‐Joint Exercises

**FIGURE 4:**
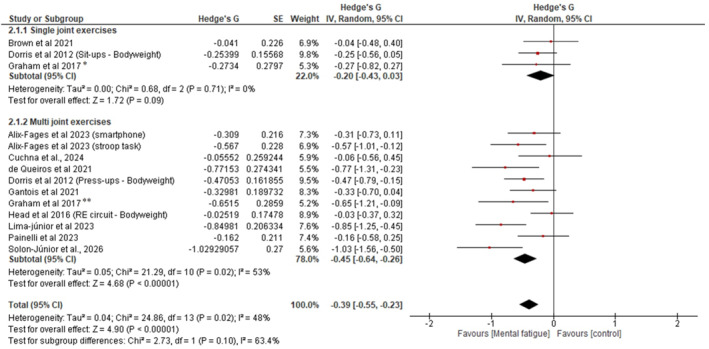
Forest plot presenting the results of the random‐effects meta‐analysis comparing MF versus control on resistance exercise volume, with subgroup analyses for single‐ and multijoint exercises.

Subgroup analysis also showed significant differences between MF and control conditions for multijoint exercises [SMD = −0.45; 95% CI = −0.64, −0.26; and *p* < 0.01; (heterogeneity: *p* < 0.05 and I^2^ = 53%)], but not for single joint exercises [SMD = −0.20; 95% CI = −0.43, 0.03; and *p* = 0.09; (heterogeneity: *p* = 0.71 and I^2^ = 0%)] as shown in Figure 4.

### 3.9 Effect of Mental Fatigue With Different Intensity Loads and Effect of Mental Fatigue According to Total Training Volume

**FIGURE 5:**
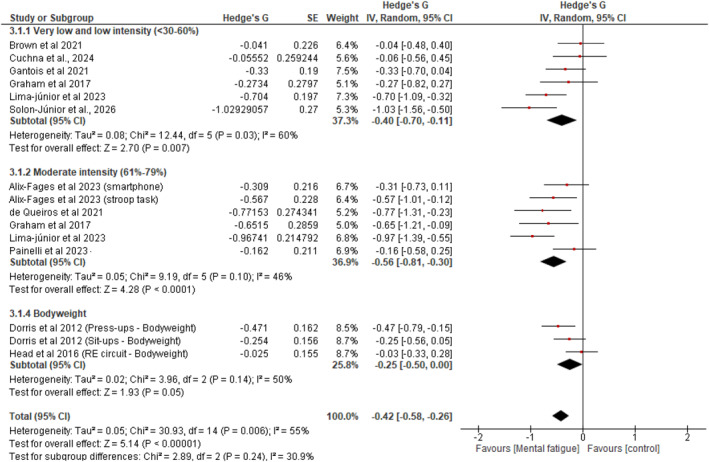
Forest plot presenting the results of the random‐effects meta‐analysis comparing MF versus control on resistance training volume, with subgroup analyses by intensity.

For subgroup analysis by exercise intensity, loads were categorized according to established resistance training intensity zones (Carvalho et al. 2022): (a) low intensity: < 60% of one‐repetition maximum (1RM), encompassing very low and low (< 30%–59% 1RM) loads; (b) moderate intensity: 60%–79% 1RM; (c) high intensity: ≥ 80% 1RM; and (d) bodyweight exercises, in which external load relative to 1RM could not be determined. As shown in Figure 5, subgroup analysis revealed significant differences between MF and control conditions for very low intensity‐load [SMD = −0.40; 95% CI = −0.70, −0.11; and *p* < 0.01; (heterogeneity: *p* < 0.05 and I^2^ = 60%)], moderate intensity‐load [SMD = −0.56; 95% CI = −0.81, −0.30; and *p* < 0.01; (heterogeneity: *p* = 0.10 and I^2^ = 46%)], and bodyweight [SMD = −0.25; 95% CI = −0.50, 0.00; and *p* = 0.05; (heterogeneity: *p* = 0.14 and I^2^ = 50%)]. The subgroup of high‐intensity load (≥ 80% 1RM) included only one study (De Lima‐Junior et al. 2024), which was insufficient to estimate a meta‐analysis. Accordingly, no pooled effect size is reported for this subgroup, and it is not included in subgroup comparisons.

Also, to examine whether the magnitude of mental fatigue effects varied according to total training volume characteristics, we conducted an additional subgroup analysis categorizing studies by relative training volume. The total training volume for each study was calculated as repetitions × sets × load intensity (%1RM). This calculation yields a relative volume metric representing cumulative training load. We could not incorporate absolute load (kilograms) into this calculation because most studies reported only the percentage of 1‐repetition maximum (%1RM) and the number of repetitions and sets completed, without reporting the actual loads lifted.

Based on the distribution of total training volume values across studies, we categorized comparisons into two subgroups: moderate total training volume (*n* = 5 comparisons from 5 studies) and high total training volume (*n* = 6 comparisons from 6 studies). The moderate total training volume subgroup demonstrated a significant negative effect of MF on resistance exercise performance (*g* = −0.37; 95% CI: −0.64, −0.10; and *p* < 0.01), with moderate heterogeneity (I^2^ = 51% and *p* = 0.07). The high total training volume subgroup also showed a significant negative effect (*g* = −0.54; 95% CI: −0.84, −0.24; and *p* < 0.01), with moderate heterogeneity (I^2^ = 52% and *p* = 0.08).

### 3.10 GRADE Summaries

**FIGURE 6:**
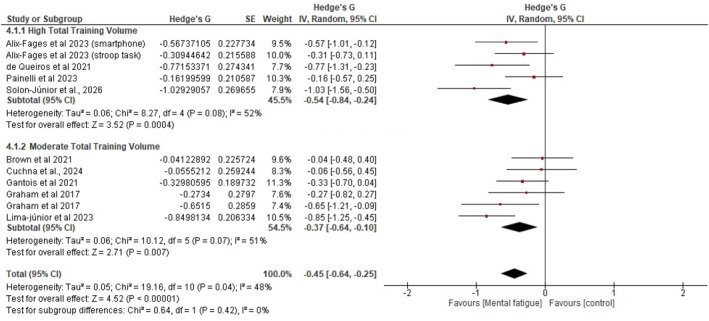
GRADE. CI: confidence interval and SMD: standardized mean difference. Explanations: (a) Different interventions of mental fatigue induction. (b) The total number of participants in this comparison is lower than the optimal information size.

**FIGURE 7:**
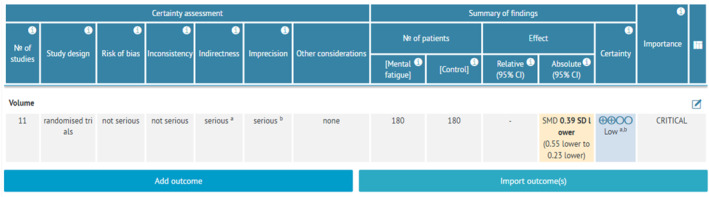
Forest plot presenting the results of the random‐effects meta‐analysis comparing MF versus control on resistance training volume, with subgroup analyses by total training volume.

Based on the GRADE assessment (Figure 6), we observed low‐quality evidence (also referred to as low certainty of evidence) supporting the negative effect of mental fatigue on resistance exercise volume. The overall quality of evidence for each GRADE domain is presented in Figure 7. The evidence was downgraded for indirectness due to heterogeneity in mental fatigue induction methods across studies (e.g., Stroop test, smartphone‐based tasks, and vigilance tests) and variations in task duration (5–52 min), which likely contribute to the observed variability in effect magnitudes. Additionally, the evidence was downgraded for imprecision as the total number of participants included in the comparison (*n* = 205) fell below the optimal information size threshold of 400 participants recommended for continuous outcomes in GRADE assessments. This limited sample size reduces confidence in the precision of the pooled effect estimate.

## 4 Discussion

The purpose of the present study was to conduct a systematic review and meta‐analysis of studies investigating the effect of prior MF on subsequent RE volume in healthy individuals. The main analysis results showed that exposure to a cognitively demanding task has a significant, small negative effect on subsequent resistance exercise volume (*g* = −0.39; 95% CI = −0.55, −0.23; and *p* < 0.01), with moderate heterogeneity in the data (*p* = 0.02 and I^2^ = 48%). The GRADE assessment revealed low‐quality evidence supporting this negative effect, indicating limited confidence in the effect estimate. Evidence was downgraded for indirectness (due to substantial heterogeneity in mental fatigue induction methods across studies, including variations in task type and duration) and imprecision (total sample size of 205 participants fell below the optimal information size threshold of 400 participants for continuous outcomes). This low‐quality rating suggests that the true effect may be substantially different from the estimated effect, and further high‐quality, adequately powered research is needed to clarify the magnitude and practical significance of these findings.

Regarding publication bias, the convergent evidence from multiple complementary methods provides reassuring support for the robustness of the primary findings. Visual inspection of the funnel plot revealed a relatively symmetric distribution, and neither Egger's regression (*p* = 0.18) nor the Begg and Mazumdar rank correlation test (*p* = 0.25) detected statistically significant asymmetry. Critically, the trim‐and‐fill procedure identified zero missing studies (*L*0 = 0), indicating that the observed funnel plot does not require adjustment and that the pooled effect estimate (*g* = −0.39) is unlikely to be substantially inflated by the “file drawer” effect. Furthermore, the test of excess significance (Ioannidis and Trikalinos 2007) yielded an O/E ratio of 0.91 (*p* = 0.73), indicating that the proportion of statistically significant findings among included studies is consistent with what would be expected given the estimated statistical power of individual studies. Collectively, these findings suggest that the body of evidence on mental fatigue and resistance exercise volume is not materially distorted by selective publication of significant results.

Several subgroup analyses also showed significant effects. For instance, multijoint exercises demonstrated significant impairment (*g* = −0.45 and 95% CI: −0.64, −0.26), whereas single‐joint exercises showed no significant effect (*g* = −0.20 and 95% CI: −0.43, 0.03), suggesting that exercise complexity modulates susceptibility to mental fatigue‐induced performance decrements. Also, in subgroup analyses across different intensity‐load zones, the magnitude of impairment appeared larger in studies employing moderate intensity (60%–79% 1RM; *g* = −0.56; 95% CI: −0.81, −0.30; and *p < 0.01*) relative to those using low‐intensity loads (*g* = −0.40; 95% CI: −0.64, −0.16; and *p = 0.02*) and bodyweight conditions (*g* = −0.25; 95% CI: −0.50, 0.00; and *p = 0.05*). Additionally, the subgroup analysis examining mental fatigue effects according to total training volume revealed significant detrimental effects in both moderate (*g* = −0.37) and high (*g* = −0.54) total training volume conditions. Collectively, these findings provide a comprehensive description of the literature examining the relationship between prior cognitive effort and RE volume, with important implications. Notably, these results build on conclusions proposed by Alix‐Fages, Grgic, et al. (in their meta‐analysis), offering new insights for trainers and RT practitioners.

The effect size observed in the present meta‐analysis (*g* = −0.39) is comparable to that reported in a prior review (Alix‐Fages, Grgic, et al. 2023b) = −0.41], despite the incorporation of additional recent studies employing diverse cognitive induction protocols. Recent studies have tended to use longer Stroop‐based paradigms (approximately 30 min), whereas earlier works often relied on shorter tasks or broader paradigms (e.g., vigilance tests or brief 5‐min Stroop tasks). Additionally, social media–based induction tasks may engage different psychobiological mechanisms than sustained inhibitory control tasks (Faro et al. 2025), which may further contribute to heterogeneity in observed effects. It is important to note that this review showed a moderate effect size (I^2^ = 48%) in the overall meta‐analysis.

Indeed, heterogeneity is common in large systematic reviews of MF and physical performance (Brown et al. 2020; Giboin and Wolff 2019). The 95% prediction interval (−0.861 to 0.081) further contextualizes the pooled estimate by incorporating between‐study heterogeneity into the uncertainty quantification. Unlike the confidence interval, which reflects uncertainty around the mean effect, the prediction interval estimates the range within which the effect of a new adequately powered study would likely fall (Riley et al. 2011). The fact that this interval crosses zero, whereas the confidence interval does not, is consistent with the low GRADE evidence rating assigned to this outcome and highlights that the magnitude of mental fatigue effects on resistance exercise volume may vary substantially across settings, populations, and cognitive induction protocols. As recently demonstrated by Borg et al. (2024), prediction intervals are substantially wider than confidence intervals in sports medicine meta‐analyses (median ratio: 3.4×) (Borg et al. 2024), and their omission may lead to overly optimistic conclusions about the reliability of pooled effects for future applications. In the present analysis, the prediction interval is approximately 3.0 times wider than the confidence interval, consistent with this pattern.

A key finding from the subgroup analyses is that the magnitude of mental fatigue's negative effect on resistance exercise outcomes appears to vary depending on the intensity‐load assessed. The findings, summarized in Figure 5, show that the negative effect of mental fatigue is greater at moderate‐intensity loads (*g* = −0.56) than at low‐intensity loads (*g* = −0.40), bodyweight (*g* = −0.25), and high‐intensity loads (*g* = −0.25). A recent study of mentally fatigued subjects (De Lima‐Junior et al. 2024) showed a 10.7% reduction in repetitions at 50% 1RM, a 13.4% reduction at 70% 1RM, and only a 6.6% reduction at 90% 1RM. Additionally, these authors (De Lima‐Junior et al. 2024) observed that subjective effort perception during repetitions was higher at moderate intensities than at low and high intensity‐load conditions. Several mechanisms may explain this intensity‐dependent relationship. High‐intensity‐load resistance exercise requires brief peak performance with minimal total work, and conscious regulation of effort does not necessitate sustained rhythm regulation, potentially allowing individuals to tolerate extremely high perceptions of exertion for short durations without meaningful performance decrements (Brown et al. 2020). In contrast, moderate‐intensity resistance exercise to volitional failure requires sustained effort regulation across a greater number of repetitions, potentially making these protocols more vulnerable to mental fatigue‐induced alterations in perceived effort and motivation (Pageaux and Lepers 2016).

Additionally, the subgroup analyses examining the effects of mental fatigue by total training volume revealed significantly greater detrimental effects in the high total training volume condition (*g* = −0.54) than in the moderate total training volume condition (*g* = −0.37). In this case, it is reasonable to suggest, at least in part, that the effect size of MF is greater in training sessions when the relative volume [repetitions × sets × load intensity (%1RM)] is elevated. It can be primarily explained by the fact that this phenomenon primarily affects perceived effort and the motivation to maintain performance (Marcora et al. 2009; Pageaux and Lepers 2016) rather than the maximum force‐generating capacity, resulting in proportional reductions in performance on tasks with higher volumes that require sustained effort. It is important to highlight that, although these findings are promising, the relative volume metric used (repetitions × sets × %1RM) may not fully capture the physiological demands of different protocols as this calculation does not account for absolute loads, rest intervals, or time under tension, which may interact differently with mental fatigue.

The finding that multijoint exercises are more susceptible to mental fatigue than single‐joint exercises aligns with theoretical predictions based on the cognitive‐motor demands of these movement patterns. Multijoint exercises (e.g., squat and bench press) impose substantially greater demands on central executive functions, including motor planning, coordination, attentional allocation, and sensorimotor integration (Seidler et al. 2004; Rozand et al. 2014), compared to single‐joint exercises (e.g., biceps curl and leg extension) that rely on simpler motor programs. Given that mental fatigue primarily impairs performance by altering cortical mechanisms that support executive function and effortful control (Marcora et al. 2009), the differential effects observed across exercise complexity are mechanistically plausible and suggest that exercise selection may modulate vulnerability to preexercise cognitive demands.

The long‐term consequences of MF, particularly regarding physical adaptations, such as hypertrophy and strength, remain an underexplored dimension of the phenomenon. To date, longitudinal research on training under mental strain is sparse. A study by Fortes et al. (2024) found that although strength (i.e., 1RM Squat) and power (i.e., Countermovement jump) were unaffected, the control group showed significantly greater improvements in the rate of force development (RFD) compared to the mentally fatigued group. This finding serves as a critical caveat for athletes who rely on explosive rapid responses during competition. Further research is essential to determine how repeated MF impacts diverse physiological markers, including muscle hypertrophy.

The present systematic review and current meta‐analysis provide a more comprehensive update of the scientific literature on cognitive effort and RE. However, some limitations must be considered. First, the low‐quality GRADE rating indicates that confidence in the precise magnitude of the effect is limited, primarily due to imprecision (total sample size below the optimal information size threshold) and indirectness (heterogeneity in mental fatigue induction methods). Second, in the body of literature examined, risk assessments of bias using the TESTEX scale identified that most studies had good methodological quality (mean score: 11.45 ± 1.34 out of 15). However, inherent limitations in blinding procedures, particularly the inability to blind participants to cognitive task conditions, may introduce performance bias. So, one way to improve the methodological quality of future studies would be to use procedures in which the researchers responsible for the study neither administer the cognitive task nor assess the outcome. Third, due to the small number of studies, another limitation is the absence of a subgroup analysis examining whether the effects of MF are lower in athletes than in physically active or recreationally trained individuals in RT. Fourth, substantial heterogeneity in mental fatigue‐induction methods (Stroop tests, vigilance tasks, and smartphone use) and task durations (5–52 min) limits the directness of the evidence and the ability to make specific recommendations about the cognitive demands encountered in real‐world training contexts.

## 5 Conclusion

Findings from this systematic review, which included 11 studies, 14 comparisons, and more than 205 subjects, provide low‐quality evidence of a small‐to‐moderate negative effect of MF on RE volume. Subgroup analyses indicate negative effects of MF in multiple but not in single‐joint exercises. However, the adverse effects of MF are more pronounced when RE involves a moderate‐intensity load (60%–79% of 1RM). To contextualize these findings for applied practice, the observed small‐to‐moderate effect sizes represent potentially significant acute reductions in training volume. In this case, practitioners who prioritize session quality and volume consistency may choose to minimize pretraining cognitive demands when circumstances allow. When cognitive activities cannot be avoided, strength and conditioning professionals may implement evidence‐based strategies to mitigate the effects of mental fatigue on the quality of resistance training. Importantly, although acute performance decrements are clearly evident, the cumulative impact of repeated mentally fatiguing training sessions on long‐term adaptations (e.g., hypertrophy) remains unclear, constituting a critical knowledge gap that warrants urgent investigation.

## Author Contributions

The design of the search strategy was performed by L.J.F.S.J. and D.L.J. and subsequently revised by L.J.F.S.J., D.L.J., and N.A. Screening on title and abstract and full text was done by N.A. and L.J.F.S.J., Data synthesis was conducted by L.J.F.S.J. and D.L.J. Figures and tables were designed by L.J.F.S.J. and DLJ. The meta‐analysis and correlation was performed by L.J.F.S.J. and revised by D.L.J. and L.S.F., T.E.S.T.E.X. was performed by L.J.F.S.J. and G.V., and subsequently revised by D.L.J., GRADE was performed by LJFSJ and DLJ. LJFSJ wrote the first draft of the manuscript which was later altered by L.S.F., S.B., S.M.M., and D.L.J., All authors read, revised, and approved the final manuscript.

## Ethics Statement

All authors declare that the systematic review complies with all ethical standards. No participants were recruited for the present study, so no consents for participation needed to be collected.

## Consent

The authors have nothing to report.

## Conflicts of Interest

The authors declare no conflicts of interest.

## Supporting information


Supporting Information S1

## Data Availability

All data generated or analyzed during this study are included in this published article (and its supplementary information files).
