# Supplementary material for: Mental Fatigue and Resistance Exercise: A Systematic Review and Meta‐Analysis Including GRADE Qualification
Source: Eur J Sport Sci. 2026 May 21;26(6):e70194. doi: 10.1002/ejsc.70194 (PMC13240521; doi:10.1002/ejsc.70194)
Supplement: Supplementary file 1 — Supporting Information S1 [file EJSC-26-e70194-s001.docx]

**Supplementary document 1 - rationale for excluding each study**

| **N** | **study** | **Title** | **Reason for exclusion** |
| --- | --- | --- | --- |
| 1 | (Mehta and Agnew 2012a) | Influence of mental workload on muscle endurance, fatigue, and recovery during intermittent static work | Not dynamic exercise |
| 2 | (Ramsay et al. 2023) | The Effect of a Mental Task Versus Unilateral Physical Fatigue on Non-Local Muscle Fatigue in Recreationally Active Young Adults. | Not dynamic exercise |
| 3 | (Alix-Fages et al. 2023a) | Mental Fatigue From Smartphone Use or Stroop Task Does Not Affect Bench Press Force–Velocity Profile, One-Repetition Maximum, or Vertical Jump Performance. | Wrong outcome variable (i.e., did not involve training volume) |
| 4 | (Alix-Fages et al. 2023b) | Mental fatigue impairs physical performance but not the neural drive to the muscle: a preliminary analysis. | Not dynamic exercise |
| 5 | (Dallaway et al. 2022) | Cognitive tasks elicit mental fatigue and impair subsequent physical task endurance: Effects of task duration and type. | Not dynamic exercise |
| 6 | (Rozand et al. 2014) | Does a Mental Training Session Induce Neuromuscular Fatigue? | Wrong intervention |
| 7 | (Proost et al. 2023) | The Impact of Mental Fatigue on a Strength Endurance Task: Is There a Role for the Movement-Related Cortical Potential? | Wrong outcome variable (i.e., did not involve training volume) |
| 8 | (Ferris et al. 2021) | Reduced Electromyographic Fatigue Threshold After Performing a Cognitive Fatiguing Task. | Wrong outcome variable (i.e., did not involve training volume) |
| 9 | (Romagnoli et al. 2024) | Can mental fatigue affect perception of barbell velocity in resistance training? | Wrong outcome variable (i.e., did not involve training volume) |
| 10 | (Harris and Bray 2021) | Mental fatigue, anticipated effort, and subjective valuations of exercising predict choice to exercise or not: A mixed-methods study | Wrong outcome variable (i.e., did not involve training volume) |
| 11 | (Wolff et al. 2018) | Increase in prefrontal cortex oxygenation during static muscular endurance performance is modulated by self-regulation strategies | Not dynamic exercise |
| 12 | (O’Brien et al. 2020) | Cardiovascular and cerebral hemodynamic responses to ego depletion in a pressurized sporting task | Not dynamic exercise |
| 13 | (Fortes et al. 2022) | Effects of social media on smartphone use before and during velocity-based resistance exercise on cognitive interference control and physiological measures in trained adults. | Wrong outcome variable (i.e., training approach with velocity loss) |
| 14 | (Lima-Junior et al. 2024) | Effects of smartphone use before resistance exercise on inhibitory control, heart rate variability, and countermovement jump | Wrong outcome variable (i.e., did not involve training volume) |
| 15 | (Hussain et al. 2020) | Cognitive stress changes the attributes of the three heads of the triceps brachii during muscle fatigue | Wrong intervention |
| 16 | (Lydakis et al. 2008) | Changes of central haemodynamic parameters during mental stress and acute bouts of static and dynamic exercise. | Not dynamic exercise |
| 17 | (Holgado et al. 2023) | Individualized Mental Fatigue Does Not Impact Neuromuscular Function and Exercise Performance | Not dynamic exercise |
| 18 | (Brown and Bray 2015) | Isometric exercise and cognitive function: an investigation of acute dose-response effects during submaximal fatiguing contractions. | Not dynamic exercise |
| 19 | (Singh et al. 2002) | Interaction of physical and mental work | Wrong outcome variable (i.e., did not involve training volume) |
| 20 | (Mehta and Agnew 2012b) | Effects of physical and mental demands on shoulder muscle fatigue | Not dynamic exercise |
| 21 | (Larsson et al. 1995) | Effects of psychophysiological stress on trapezius muscles blood flow and electromyography during static load. | Not dynamic exercise |
| 22 | (Stock et al. 2011) | The effects of diverting activities on recovery from fatiguing concentric isokinetic muscle actions | Wrong outcome variable (i.e., number of standardized repetitions) |
| 23 | (Staiano et al. 2023) | Mental fatigue impairs repeated sprint and jump performance in team sport athletes. | Wrong outcome variable (i.e., did not involve training volume) |
| 24 | (Magnuson et al. 2021) | Development and recovery time of mental fatigue and its impact on motor function | Wrong outcome variable (i.e., did not involve training volume) |
| 25 | (Cruz-Montecinos et al. 2018) | Influence of a self-regulated cognitive dual task on time to failure and complexity of submaximal isometric force control. | Not dynamic exercise |
| 26 | (Dıaz-Garcıa et al., 2024) | Brain Endurance Training Improves and Maintains  Chest Press and Squat Jump Performance  When Fatigued | Wrong design |
| 27 | (Marcos-Frutos et al. 2025) | The Impact of Rhodiola Rosea Extract on Strength Performance in Alternative Bench-Press and Bench-Pull Exercises Under Resting and Mental Fatigue Conditions: A Randomized, Triple-Blinded, Placebo-Controlled, Crossover Trial | Wrong intervention |
